# Debate. How should steps per day be reported--a proposal using data from Africa

**DOI:** 10.1186/1479-5868-9-7

**Published:** 2012-02-06

**Authors:** Ian Cook

**Affiliations:** 1 Physical Activity Epidemiology Laboratory, University of Limpopo (Turfloop Campus), P.O. Box 459, Fauna Park, Polokwane 0787, Limpopo Province, South Africa

**Keywords:** Pedometer, Physical activity, Africa

## Abstract

Recent reviews published in the *IJBNPA *provide more definitive ranges of ambulatory activity usually found in four general age groups, and how step data relates to public health guidelines. Of note in these reviews was the lack of data from Developing Countries. Apart from the lack of published data, available data was not presented in a format conducive to inclusion in the reviews. Consequently, this paper presents available pedometry data from Africa, such that it is easily available for inclusion in future comparisons. Also addressed is the need to consider adjusting prevalence data according to standard population age structures.

## Correspondence

### Introduction

Recently, three reviews were published in the *IJBNPA *which address the need to update accumulating evidence, relating to step-based recommendations, with evidence-based physical activity guidelines [1-3]. What is apparent in the reviews is the lack of pedometry data from Developing Countries, specifically Africa. The primary reason for the absence of African data in the reviews is simply the dearth of reported African data. However, the published data that is available [4,5] could not be included [2] because of the reporting format in the original papers. The unadjusted step data was reported either as mean(sd) by age quartile [4] or mean(sd) by step quartile [5] and adolescent/adult and adult/elderly age categories overlapped substantially in the first and last quartiles, respectively. With regards to the reviews [1-3] a possible area of concern is that the reported prevalence statistics, especially for adults, do not appear to have been adjusted to standard populations, making comparison across moderate to large scale surveillance studies difficult. Consequently, the purpose of this paper is to present the available step data from African samples such that the data can be used in future reviews and to encourage researchers especially from Developing Countries to report step data in a more standardised format.

## Methods

The methodology for the two cross sectional studies conducted in a rural African population [6-8] is described in detail elsewhere [4,5]. The age range is 14-96 years and the average daily step range is 1048-35534 steps [4,5]. The first smaller study (n = 121) conducted in 2003-2004 on females only, used the Yamax Digiwalker SW-401 pedometer [4]. The second larger study (n = 789) collected data from both sexes in 2005-2007 using the New Lifestyles NL-2000 pedometer [5].

The suggested reporting format contains 8 step levels (< 2500, 2500-4999, 5000-7499, 7500-9999, 10000-12499, 12500-14999, 15000-17499, ≥17500) [9]. Unlike samples from Developed Countries which tend to require more sedentary categories, samples from Developing Countries often report very high step values [5] due to the dependence on subsistence activities which include substantial amounts of walking [10]. Therefore, an upper limit of 17500 steps was chosen [11]. The recent reviews used age ranges for children, adolescents, adults and the elderly of 6-11.9, 12-19.9, 20-65 and > 65 years, respectively [1-3]. However, in the current data sets, because the sampling includes adolescents from 14 to 19.9 years and the age distribution within the adult and elderly age categories are skewed, sub-categories were created.

The reporting format is included as supplementary material in two spreadsheet files. The first file [see Additional file 1] consists of three tables containing descriptive data and step data across age, sex and step index for both data sets. The descriptive statistics include mean(sd), percentages and *n*. The second file [see Additional file 2] uses a simple methodology [12] to adjust prevalence statistics to specified populations using four- and eight category step indices across standard demographic age ranges. Where necessary, data were analysed using appropriate statistical software (SPSS Inc. PASW: Release 18.0.3 SPSS Corp, Chicago, Il, 2010). Significance was set at *p *< 0.05.

## Results and discussion

Because the data [4,5] was collected in one field site [6-8], the data sets were checked for duplicates. Twenty seven duplicates were identified, all female with 24, 2 and 1 in the adult, adolescent and elderly categories, respectively. The duplicate group tended to be slightly older and heavier and walked less (Age: delta = +0.9 years, *p *= 0.018; BMI: delta = +4.2 kg.m^-2^, *p *= 0.048; Ambulation: delta = -848 steps, *p *= 0.437). Excluding duplicate data [5] changed average step data by < 85 steps and the crude step index prevalence changed by ≤0.7%. Consequently, duplicate data was included in the analysis.

Step values for adolescents fell within the ranges observed for children from Developed Countries [1] with no or little decline over the 14-19.9 year age range. Adults demonstrated a steady decline over the 20-65 year age range although the mean values at all age ranges were well above the minimum recommendation of 7000-8000 steps/day [2]. The adult age range spans 45 years and should reflect the populations' age distribution for that category. For instance, the adult crude and adjusted prevalence for ≥10000 steps for one of the data sets [5] are 65.8% and 68.8%, respectively using the INDEPTH age structure [13] as the standard. In the elderly, > 65 years, > 20% achieve ≥10000 steps/day, and mean step values were in the upper end of those observed for Developed Countries [3]. The generally elevated step values for all age groups reflect the greater reliance on subsistence living and active transport and are a useful comparison for future studies.

## Conclusions

Researchers in Developing Countries, especially Africa, are strongly encouraged to collect and publish step count data. Without such data there is the possibility of bias toward values obtained in Developed Countries. Importantly, systematic reviews can only include published data that is appropriately formatted.

## Competing interests

The author declares that they have no competing interests.

## Supplementary Material

Additional file 1 **Sheet 1--Table 1. Age and body mass index of free-living rural black South Africans by step index**. **Sheet 2**--**Table 2**. Free-living ambulatory profile of rural black South African women (n = 121, 19-56 years) by age and step index (2003-2004). **Sheet 3**--**Table 3**. Free-living ambulatory profile of rural black South Africans (n = 789, 14-96 years) by gender, age and step index (2005-2007). Click here for file

Additional file 2 **Sheet 1--Age adjustment for 4-category step index prevalence**. **Sheet 2**--Age adjustment for 8-category step index prevalence. **Sheet 3**--Age profile chart. **Sheet 4**--Instructions, references. Click here for file
